# Knowledge regarding antibiotic use among students of three medical schools in Medellin, Colombia: a cross-sectional study

**DOI:** 10.1186/s12909-020-1934-y

**Published:** 2020-01-28

**Authors:** Luis Felipe Higuita-Gutiérrez, Valentina Molina -Garcia, Jenifer Acevedo Guiral, Liceth Gómez Cadena, Gustavo Eduardo Roncancio Villamil, Judy Natalia Jiménez Quiceno

**Affiliations:** 1 Facultad de Medicina Universidad Cooperativa de Colombia, Escuela de Microbiología, Universidad de Antioquia, Medellín, Colombia; 2 Escuela de Microbiología, Universidad de Antioquia, Medellín, Colombia; 3 Facultad de Medicina Universidad Pontificia Bolivariana, Clinica Cardio VID, Grupo Microba, Universidad de Antioquia, Medellín, Colombia; 4 Escuela de Microbiología, Grupo MICROBA, Universidad de Antioquia, Medellín, Colombia

**Keywords:** Antibiotic resistance, Education medical, Prescription drug misuse

## Abstract

**Background:**

The objective of the present study was to describe the knowledge regarding the antibiotic therapy of students of three medical schools in Medellín, Colombia.

**Methods:**

The study population comprised medical students who were enrolled in three universities. The instrument contained questions regarding their current academic term, the university, the perceived quality of the education received on antibiotic therapy and bacterial resistance, and specific questions on upper respiratory tract infections, pneumonia, urinary tract infections, and skin and soft tissue infections. The information was analyzed by calculating frequencies and measures of dispersion and central tendency. Knowledge regarding the treatment for each type of infection was compared using the Mann–Whitney U test and the Kruskal–Wallis H test.

**Results:**

We included 536 medical students, of which 43.5% students consider that the university has not sufficiently trained them to interpret antibiograms and 29.6% students consider that the quality of information received on the subject at their university ranges from regular to poor. The mean score for knowledge regarding antibiotic therapy for upper respiratory tract infections was 44.2 (9.9) on a scale from 0 to 100. The median score with regard to the treatment of pneumonia was 52.9 (14.7), that of urinary tract infection was 58.7 (14.8), and that of skin and soft tissue infections was 63.1 (19.4). The knowledge regarding antibiotic therapy for upper respiratory tract infections, pneumonia, and urinary tract infection does not improve with the academic term, the university, or perceived quality of the education received.

**Conclusion:**

A large proportion of medical students perceive that the training received from the university is insufficient with regard to antibiotic use and bacterial resistance, which is consistent with the limited knowledge reflected in the selection of antibiotic treatment for respiratory, urinary tract, and skin and soft tissue infections. Overall, the situation was identical among all universities, and it did not significantly increase with the completion of an academic term.

## Background

Antibiotics are fundamental drugs in modern medicine because they have significantly decreased mortality due to infectious diseases and improved survival as well as have been essential in preventing or treating infections that can occur in patients who are receiving chemotherapy treatments, who have chronic diseases, or who have undergone complex surgeries such as organ transplants, joint replacements, or cardiac surgery [1, 2].

The antibiotic resistance crisis has been attributed to several aspects, among which special attention should be paid to the overuse and misuse of these medications; these aspects have led to an antibiotic resistance crisis and to a serious issue of public health that constitutes a threat to all advances achieved by modern medicine. Infections by resistant bacteria do not respond to standard antibiotic treatment and result in an increased number of morbidity and mortality cases and excess healthcare cost. The easy dissemination of these bacteria between countries, due to international travel, compromises global public health [3, 4].

It is estimated that resistant bacteria cause approximately 25,000 deaths in Europe and at least 2 million infections in the United States annually. There are few reliable estimates for developing countries. However, there may be a greater impact of antimicrobial resistance owing to the increase in infectious diseases and restricted access to new antibiotics [5]. Moreover, the emergence of new resistance mechanisms that complicate the treatment of common infectious diseases, such as pneumonia, tuberculosis, and septicemia, or sexually transmitted diseases, such as gonorrhea, is common [3].

However, several doctors believe that bacterial resistance is a rare issue in daily clinical practice. In addition, they misrepresent the evidence that links inappropriate antibiotic prescriptions with bacterial resistance, thereby leading to more serious consequences for their patients, such as longer hospital stay, more invasive treatments, or death [6]. One of the consequences of considering the issue as not very serious or of little relevance for routine clinical practice is the lack of interest in prudently using antibiotics. For instance, regarding the aforementioned aspect, one study revealed that 23% of antibiotic prescriptions in the United States are inaccurate [7]. In Saudi Arabia, > 46% of prescriptions are written for clinical conditions for which antibiotics are not indicated [8], and in Colombia, a study revealed that between 29.2 and 67.4% of the doctors surveyed have incorrect knowledge regarding antibiotic prescription [9].

In this context, the World Health Organization (WHO) advocates to implement strategies that allow the next generation of doctors to be better prepared to appropriately use antibiotics and combat bacterial resistance. Consistent with the abovementioned, the aim of the present study was to describe the knowledge regarding antibiotic therapy of students of three medical schools in Medellín, Colombia. The evaluation of this aspect will facilitate understanding the level of knowledge of these students and guiding future interventions.

## Methods

Type of study: cross-sectional descriptive

Subjects of study and sample: the study population comprised medical students who were enrolled in 2018 in three universities in the city of Medellin.

The sample size was calculated based on a reference population of 3324 medical students in the three universities, an expected deviation of 12 points on the scale that assesses the knowledge regarding antibiotic use for each type of infection, a confidence level of 95%, sampling error of 1%, and sampling correction of 10%.

The sampling unit was the university and the selection of the participants was performed at convenience, considering the inclusion of students of all semesters visiting all the classrooms and inviting all those who willingly wanted to participate. The inclusion criteria were defined as being a medical student from one of the three universities included in the study of any sex and age. Students who rejected voluntary participation in the study, who demanded remuneration, and participants who had not completed > 10% (14 items) of the questions of the survey were excluded.

Information-gathering instrument: An instrument designed by an infectious disease physician, a doctor in molecular epidemiology with experience in bacterial resistance research, and a microbiologist with a master’s degree in education was used to collect information. The questionnaire was developed and administered in Spanish to the students. The instrument was divided into five sections: The first section contained questions regarding their current academic term (Semester 1 to 5: Basic, Semester 6 to 10: Clinics, and Semester 11 to 12: Internship), the university, and the perceived quality of the education received on antibiotic use and bacterial resistance. The other sections include specific questions on upper respiratory tract infections (8 questions), pneumonia (4 questions), urinary tract infections (7 questions), and skin and soft tissue infections (3 questions). To ensure that the score obtained in each section is comparable, summations of the result obtained in each of them were calculated and the four scores were generated, which were scored between 0 (worst) and 100 (best) using the following formula:

$$ \mathrm{Formula}=\frac{\mathrm{Score}\ \mathrm{obtained}\ \mathrm{in}\ \mathrm{section}-\mathrm{minimum}\ \mathrm{score}\ \mathrm{possible}\ \mathrm{in}\ \mathrm{section}}{\mathrm{Maximum}-\mathrm{minimum}\ \mathrm{score}\ \mathrm{in}\ \mathrm{section}}\times 100 $$

### Procedure

For the collection of information, educational institutions were contacted, and the project was presented to the students who voluntarily participated in the study and completed an anonymous survey. Interviewer, instrument, and respondent biases were controlled during the collection of information. The interviewer conducted a training that included a protocol with operational definitions of the variables and guidelines on the fieldwork. A pilot test and validity of appearance was applied to the instrument. Respondents were guaranteed confidentiality and anonymity.

### Analysis of the information

The information was analyzed by calculating absolute and relative frequencies for the qualitative variables and measuring position, dispersion, and central tendency for the quantitative variables. Knowledge about the treatment of each type of infection, according to the perceived quality of education received, was compared using the Mann–Whitney U test and the Kruskal–Wallis H test, after verifying the non-fulfillment of the assumption of normality evaluated using the Kolmogorov–Smirnov test with Lilliefors correction. Three conditions were used to evaluate the potential confounding factors: i) the factor was not an intermediate step in the causal event horizon; ii) the variable might reveal an association with the study group or illness; and iii) the variable might reveal an association with one type of infection. Therefore, the quantification of confounding factors was performed using multiple linear regression models. All analyses were performed in SPSS Version 25.0, and *p*-values of < 0.05 were considered significant.

### Ethical aspects

The study was approved by the ethics committee of Universidad Cooperativa de Colombia according to item number 023–2018, through record N0.001.

## Results

We included 536 medical students, mostly women (60.4%), aged 16–49 years, from the first semester through internship. When enquired regarding the perceived quality of the education received on antibiotic use and bacterial resistance, 43.5% of the medical students considered that the university has not sufficiently trained them to interpret antibiograms. Furthermore, 46% of the students considered that they received insufficient training with regard to switching from intravenous (IV) to oral antibiotics, and 21.4% of the students considered themselves inadequately trained to find reliable sources of information. Typically, 29.6% of the students considered that the quality of information received on the subject at their university ranged from regular to poor (Table 1).

**Table 1:** The academic term, university, and perceived quality of the education received regarding antibiotic therapy

		Number	Percent
Academic term	Basic	218	40.7
	Clinical areas	152	28.4
	Internship	166	31.0
University	U1	215	40.0
	U2	170	31.6
	U3	153	28.4
The university adequately prepares you to…	… know when to initiate antibiotic treatment	473	89.9
	… select the antibiotics for each infection	423	80.4
	… understand the basic resistance mechanisms	440	83.7
	… interpret antibiograms	296	56.5
	… find reliable sources of information	412	78.6
	… switch from IV to oral antibiotics	282	54.0
Evaluation of information received on the subject	Not received	32	6.0
	Average/Poor	157	29.6
	Good	258	48.7
	Excellent	83	15.7
Has experience in research or education regarding antibiotic and/or bacterial resistance		296	55.0

Basic: Includes students from the first to fifth semesters

Clinical areas: Includes students from the sixth to tenth semesters

Internship: Includes students from the eleventh and twelfth semesters

For confidentiality, the three universities included were coded as U1, U2, and U3

IV: Intravenous

### Upper respiratory tract infections

**Fig. 1:**
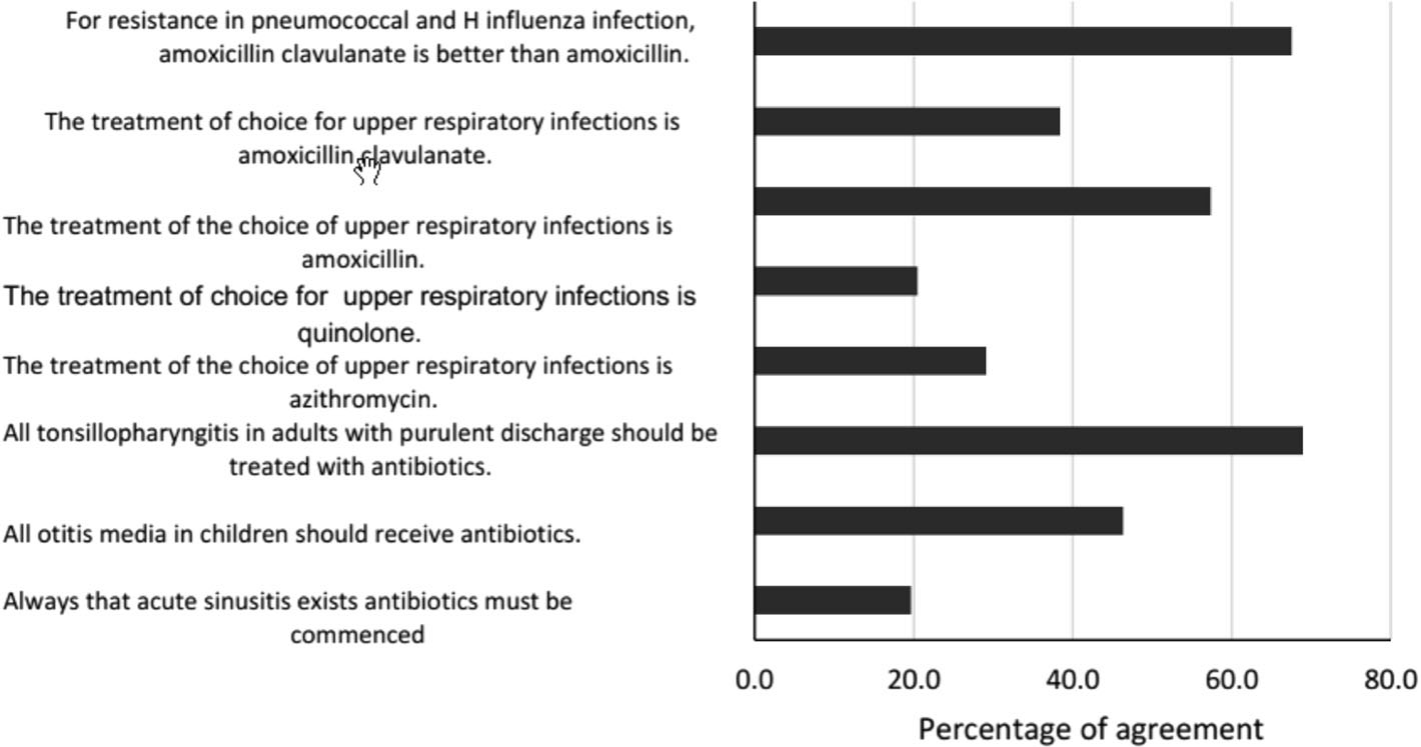
Relative frequency of responses for upper respiratory tract infections

Regarding antibiotic use for the treatment of upper respiratory tract infections, 46.3% (*n* = 242) of the medical students considered that each case of otitis media in children should be treated with antibiotics and 29.1% (*n* = 150) of the students stated that the treatment of choice for these infections should be azithromycin (Fig. 1). The mean (SD) score for knowledge regarding antibiotic therapy for these type of infections was 44.2 (9.9) on a scale from 0 to 100 (Table 2), and bivariate analysis showed that the knowledge does not improve with the academic term, university, or perceived quality of the education received (Table 3).

**Table 2:** Knowledge profile regarding antibiotic therapy according to the type of infection

	Mean (SD)	Me (IQR)	Minimum	Maximum
Treatment for upper respiratory tract infections	44.2 (9.9)	42.9 (39.3–50.0)	14.3	71.4
Treatment for pneumonia	52.9 (14.7)	50.0 (41.7–66.7)	0.0	91.7
Treatment for urinary tract infections	58.7 (14.8)	61.9 (52.4–66.7)	9.5	95.2
Treatment for skin and soft tissue infections	63.1 (19.4)	66.7 (55.6–77.8)	0.0	100.0

*SD* Standard deviation

*Me* Median

*IQR* Interquartile range

**Table 3:** Knowledge regarding antibiotic therapy for infections according to the study factors

		Upper respiratory infection Me (IQR)	Pneumonia Me (IQR)	UTI Me (IQR)	Skin and soft tissue infection Me (IQR)
Academic term	Basic	46.4 (35.7–50.0)	50.0 (41.7–58.3)	57.1 (42.9–66.7)	55.6 (44.4–77.8)
	Clinical areas	42.9 (35.7–50.0)	50.0 (41.7–66.7)	61.9 (52.4–71.4)	66.7 (44.4–77.8)
	Internship	46.4 (39.3–53.6)	50.0 (50.0–66.7)	61.9 (52.4–71.4)	66.7 (55.6–77.8)
	*P* value	0.549	0.051	0.001**	0.002**
University	U1	42.9 (35.7–50.0)	50.0 (41.7–58.3)	57.1 (42.9–66.7)	55.6 (44.4–77.8)
	U2	42.9 (35.7–50.0)	50.0 (50.0–66.7)	61.9 (52.4–66.7)	66.7 (55.6–77.8)
	U3	46.4 (39.3–53.6)	50.0 (50.0–66.7)	61.9 (52.4–71.4)	66.7 (44.4–77.8)
	*P* value	0.115	0.100	0.000**	0.002**
Knowing when to initiate antibiotic treatment	No	46.4 (39.3–50.0)	58.3 (50.0–66.7)	61.9 (52.4–71.4)	55.6 (44.4–77.8)
	Yes	42.9 (35.7–50.0)	50.0 (41.7–66.7)	61.9 (52.4–66.7)	66.7 (55.6–77.8)
	*P* value	0.535	0.323	0.481	0.109
Selecting the antibiotics for each infection	No	46.4 (39.3–50.0)	50.0 (41.7–66.7)	61.9 (52.4–71.4)	55.6 (44.4–77.8)
	Yes–	42.9 (35.7–50.0)	50.0 (41.7–66.7)	61.9 (52.4–66.7)	66.7 (55.6–77.8)
	*P* value	0.574	0.456	0.693	0.234
Understanding the basic resistance mechanisms	No	46.4 (39.4–53.6)	58.3 (50.0–66.7)	61.9 (57.1–71.4)	55.6 (44.4–77.8)
	Yes	42.9 (35.7–50.0)	50.0 (41.7–66.7)	57.1 (47.6–66.7)	66.7 (55.6–77.8)
	*P* value	0.107	0.028*	0.041*	0.804
Interpreting antibiograms	No	46.4 (39.3–50.0)	50.0 (41.7–66.7)	61.9 (52.4–71.4)	66.7 (55.6–77.8)
	Yes	42.9 (35.7–53.6)	50.0 (41.7–58.3)	57.1 (47.6–66.7)	55.6 (44.4–77.8)
	*P* value	0.909	0.107	0.055	0.002**
Finding reliable sources of information	No	46.4 (39.3–50.0)	50.0 (41.7–66.7)	61.9 (52.4–66.7)	55.6 (44.4–77.8)
	Yes	42.9 (39.3–50.0)	50.0 (41.7–66.7)	61.9 (47.6–66.7)	66.7 (44.4–77.8)
	*P* value	0.384	0.515	0.393	0.691
Switching from IV to oral antibiotics	No	42.9 (39.3–50.0)	50.0 (50.0–66.7)	61.9 (52.4–71.4)	66.7 (44.4–77.8)
	Yes	42.9 (35.7–50.0)	50.0 (41.7–58.3)	57.1 (47.6–66.7)	55.6 (44.4–77.8)
	*P* value	0.329	0.131	0.002**	0.664
Research or education	No	46.4 (39.3–53.6)	50.0 (41.7–66.7)	61.9 (52.4–66.7)	66.7 (55.6–77.8)
	Yes	42.9 (35.7–50.0)	50.0 (41.7–66.7)	57.1 (47.6–66.7)	66.7 (44.4–77.8)
	*P* value	0.400	0.697	0.259	0.869

Basic: Includes students from the first to fifth semesters

Clinical areas: Includes students from the sixth to tenth semesters

Internship: Includes students from the eleventh and twelfth semesters

For confidentiality, the three universities included were coded as U1, U2, and U3.

*IV* Intravenous

*UTI* Urinary tract infection

*Me* Median

*IQR* Interquartile range

* *P* value < 0.05, ** *P* value < 0.01

### Pneumonia

**Fig. 2:**
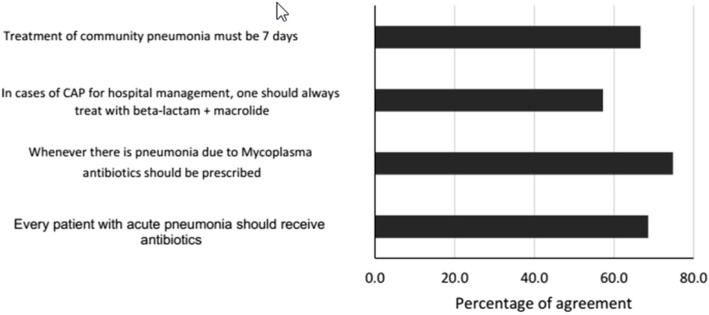
Relative frequency of responses for pneumonia

In the treatment of pneumonia, 68.5% (*n* = 351) of the students stated that all patients with acute pneumonia should receive antibiotics, whereas 74.8% (*n* = 377) of the students indicated that they must be prescribed in case of pneumonia due to *Mycoplasma* spp., (Fig. 2). The median score for these types of infections was 52.9 (14.7) (Table 2), and the knowledge does not improve with the academic term, university, or perceived quality of the education received (Table 3).

### Urinary tract infections

**Fig. 3:**
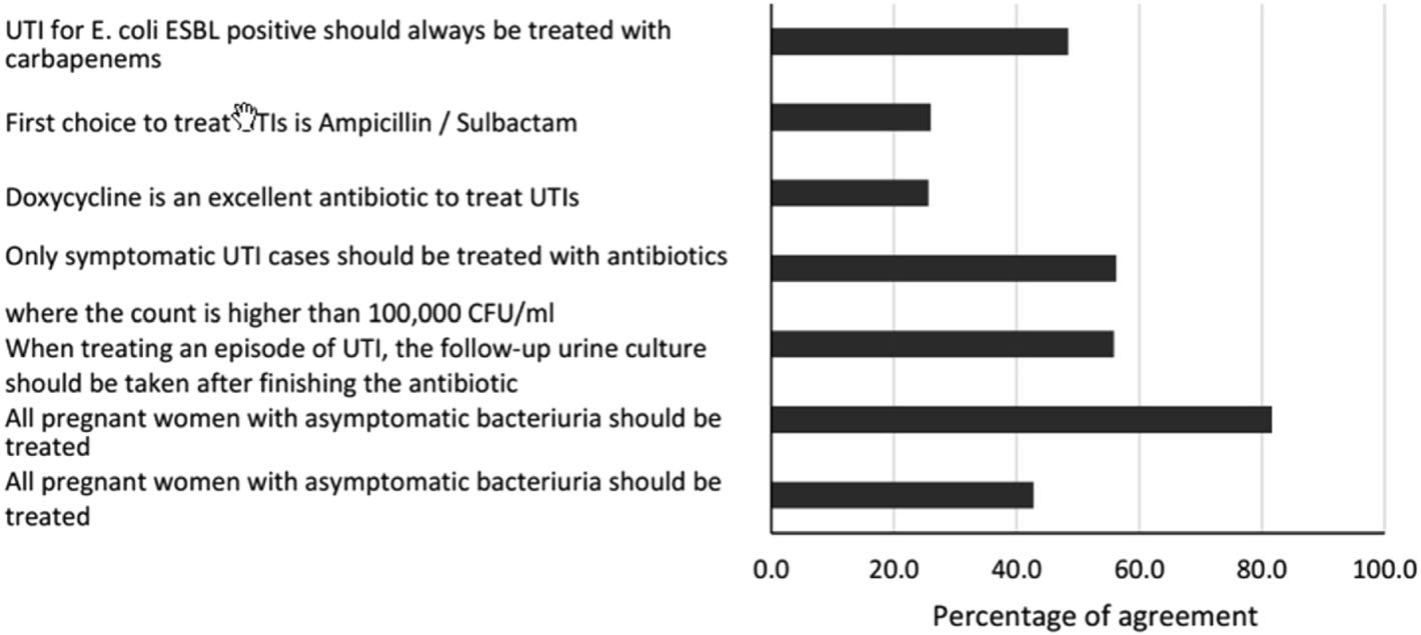
Relative frequency of responses for urinary tract infections

Regarding urinary tract infections (UTIs), 42.8% (*n* = 216) of the medical students stated that every asymptomatic bacteriuria in women with diabetes should be treated. Moreover, they stated that the follow-up urine culture in patients undergoing treatment for UTI should be performed after the completion of the antimicrobial therapy, and 55.8% (*n* = 281) and 26% (*n* = 130) of the students stated that the first choice of treatment for UTI should be ampicillin/sulbactam (Fig. 3). The mean score for this index was 58.7 (14.8) (Table 2), and knowledge slightly improves with the academic term and university (Table 3). In the multivariable analysis, only the university and switching from IV to oral antibiotics showed an association with Knowledge regarding antibiotic use.

### Skin and soft tissue infections

**Fig. 4:**
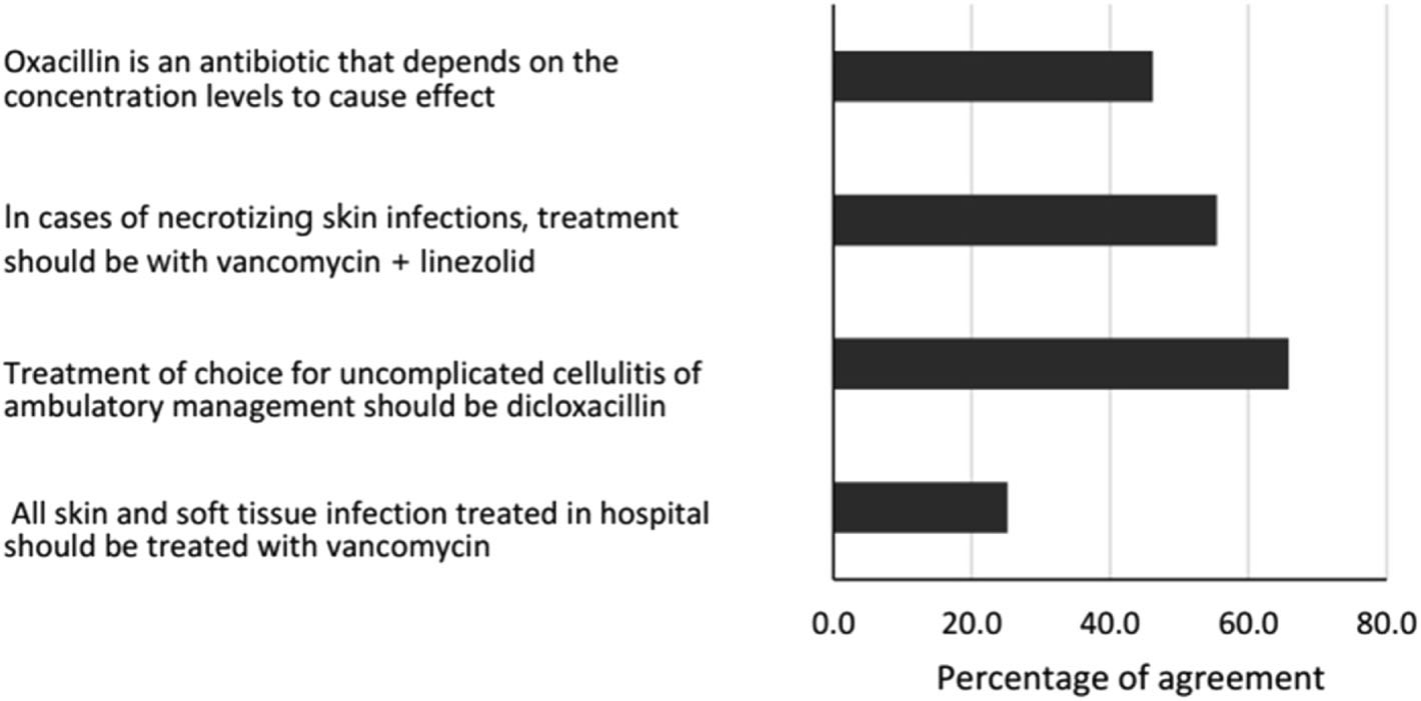
Relative frequency of responses for skin and soft tissue infections

In reference to skin and soft tissue infections, 25.2% (*n* = 126) of the students reported that all skin and soft tissue infections requiring hospital management should receive vancomycin, and 55.4% (*n* = 276) of the students stated that in necrotizing skin infections, treatment should be vancomycin in combination with linezolid (Fig. 4). The score for this index was the highest among all the ones evaluated at 63.1 (19.4) (Table 2), and it significantly improves with the academic term and university (Table 3). In the multivariable analysis, only the academic term showed an association.

## Discussion

The present study revealed that medical students exhibit poor knowledge regarding antibiotic use, with the scores between 44.2 (9.9) and 63.1 (19.4) points. Typically, students perceive that training received from the university regarding the topic is insufficient. In this regard, it is important to remember that the WHO has highlighted the importance of improving the training of undergraduate students with regard to antibiotic use as one of the main strategies to preserve their effectiveness of antibiotics [10]. However, the results of the present study, along with those reported for students from the United States [11], Spain [12], and seven other European countries [13] reflect that education regarding this topic remains inadequate.

The interpretation of antibiograms was highlighted among the topics in which the students considered that they received insufficient training by the university, with 43.5% of the students stating this. This finding is similar to that of an investigation conducted in students in China, where the frequency of dissatisfaction with the education received to interpret antibiograms was 71.7% [14]. The interpretation of antibiograms is a fundamental competence for trainee doctors because it guides the detection of the new resistance mechanisms, knowledge of the epidemiology in a defined geographical area, and choice of antimicrobial treatment. However, the interpretation of an antibiogram is a complex exercise that involves appropriate knowledge, for instance, the knowledge that there are antibiotics that are only slightly affected by the resistance mechanisms and hence are reported as sensitive in inhibitory tests in cases when they are resistant is crucial. A classic example is the false sensitivity of *Salmonella* spp. to ciprofloxacin and levofloxacin, despite these isolates being resistant to nalidixic acid. Similarly, the knowledge regarding the false sensitivity of *Staphylococcus aureus* to amikacin and tobramycin, when the organism is resistant to gentamicin, is important [15]. Failure to recognize these characteristics has an impact in the choice of therapy—it leads to therapeutic failure, omits the reporting of new resistance mechanisms and increases costs owing to the requirement of specialized diagnostic tests. Therefore, teaching in this field constitutes a challenge for the city medical schools. Nevertheless, it is necessary to complement these actions by encouraging their mission as promoters of health education, particularly to ensure that patients follow medical recommendations and adhere to therapies.

With regard to knowledge about the treatment of specific infections, it was found to be low for respiratory tract infections due to a tendency toward the indiscriminate azithromycin use, otitis treatment selection in children, and antibiotic use in cases of acute pneumonia. This finding is consistent with previous studies conducted in practicing physicians that found that 45–64.2% [16] of antibiotic prescriptions for patients with respiratory tract infections are inadequate [17]. Particularly, in medical students, it has been observed that 18.1% considered that antibiotics are useful for the treatment of viral respiratory tract infections [18]. These findings demonstrate that it is crucial to improve the knowledge of the treatment of respiratory tract infections in trainee physicians, because these infections are among the 10 main causes of morbidity and mortality in the general population and among the first 3 causes in the pediatric population [19]; moreover, cases of pneumonia are the leading cause of death due to infectious diseases [20]. A lack of improvement of the knowledge contains two implications. On the one hand, antibiotic prescriptions for cases in which they are not indicated contribute to the selection pressure for resistant microorganisms. On the other hand, appropriate treatment is delayed, contributing to morbidity and mortality.

With regard to UTIs, the mean score for this ratio was 58.7 (14.8) points, with a high proportion of students stating that all asymptomatic urinary infections in women with diabetes must be treated and that the first choice of treating a UTI must be ampicillin/sulbactam. This finding is consistent with that of another investigation conducted in which 47.3% of the students do not identify the appropriate UTI therapy [14]. In addition to errors pertaining to the appropriate therapy, research conducted on practicing physicians found that only 41% of antibiotic prescriptions for these types of infections are written according to the recommended dosing, interval, and duration [21]. It has been described that in up to 96% of cases, antibiotics that are not indicated for UTIs in pregnant women are being prescribed [22]. Errors in antibiotic prescriptions for these types of infections is a crucial issue, considering that UTIs are one of the most common causes of doctor visits at the primary care level, affecting approximately 150 million individuals annually worldwide [23]. In the United States, these cases are the cause for 0.7% of all outpatient visits. It is estimated that annually, 7 million women seek medical care due to UTIs [24], and 15% of all antibiotics prescribed in outpatient clinics are directed toward treating these infections [25]. Furthermore, in the case of pregnant women, these medicines can present deleterious effects on the fetus [22].

The knowledge regarding the treatment of skin and soft tissue infections showed a mean score of 63.1 (19.4), with a tendency for vancomycin use in nosocomial cases and in necrotizing infections. The frequencies of these infections have presented a dramatic increase between 2000 and 2004, with values reaching 29% of total hospitalization cases. Moreover, they are attributed for 6.3 million visits to the doctor annually. An important proportion of this frequency is linked to the appearance of community acquired infections by methicillin-resistant *S. aureus* (MRSA) [26]. With the appearance of MRSA, vancomycin use has become popular, which could explain the students’ tendency to prescribe this antibiotic. However, the use and abuse of this drug has led to cases of vancomycin-resistant *S. aureus*. Although resistance to vancomycin is less critical than predicted because the strains identified are not pan-resistant and are susceptible to commonly used antibiotics, such as trimethoprim–sulfamethoxazole or linezolid, it is of utmost importance to insist on the prudent use of these antimicrobials at their early stages of formation [27].

Interventions directed to the improvement of antibiotic use have traditionally been focused on clinicians and pharmacists [28, 29] or have been restricted to evaluating the effects of programs to control infections associated with healthcare [30]. Among medical students, interventions are inadequate, despite the potential to exert substantial effects in them because they have not yet developed erroneous prescription habits [31]. Some interventions of this kind can be found at universities in the United States [11, 32, 33]. One of the main measures that can be undertaken to improve the knowledge and ability for the appropriate medication use among medical students is the personal drug selection method. This method, suggested by the WHO [34] has successfully been applied in different countries such as Nepal [35] and Japan [36]. Similarly, Silverberg et al. [37] conducted a review of recent literature in which they identified 48 articles, distributed worldwide, with different teaching methodologies on antibiotic administration in undergraduate and postgraduate medical education, and although that study showed that medical schools worldwide are implementing interventions on this topic, a rigorous evaluation of interventions is required to determine if such efforts have indeed been effective. Such interventions and evaluation could provide a basis on which to focus micro- and macro-curricular academic changes for local universities.

The possible limitations to this study include failure to consider the study plans of medical schools regarding antibiotic use and bacterial resistance. The information gathered was based on self-reporting questionnaire, and because three of the six universities in the city were included, external validity was compromised.

## Conclusion

Despite the abovementioned limitations, the study provides a conclusion that a large proportion of medical students perceive that the training received from the university is insufficient with regard to antibiotic use and bacterial resistance, which is consistent with the limited knowledge reflected in the selection of antibiotic treatment for respiratory, urinary tract, and skin and soft tissue infections. Overall, the situation is identical among all universities, and it did not significantly increase with the completion of an academic term. Considering this situation, although it is crucial to act in different sectors, it is evident that education with regard to adequate antibiotic prescription as well as infection control and prevention is the basis for solving the issue.

## Data Availability

The datasets analysed during the current study are available from the corresponding author on reasonable request.

## Abbreviations

IV: Intravenous
U: University
UTI: Urinary tract infections
